# The origins of health and disease: the influence of maternal diseases and lifestyle during gestation

**DOI:** 10.1186/1824-7288-39-7

**Published:** 2013-01-23

**Authors:** Lucetta Capra, Giovanna Tezza, Federica Mazzei, Attilio L Boner

**Affiliations:** 1 Department of Reproduction and Growth, Section of Pediatrics, Azienda Ospedaliera Universitaria Sant’Anna Ferrara, Ferrara, Italy; 2 Department of Life Sciences and Reproduction, Section of Pediatrics, University of Verona, Policlinico G.B. Rossi, Verona, Italy

**Keywords:** Diseases in pregnancy, Depression, Psychosocial stress, Epilepsy, Diabetes, Asthma, Sleep disorders, Maternal diet, Pollutants, Folic acid

## Abstract

According to the Barker hypothesis, the period of pregnancy and the intrauterine environment are crucial to the tendency to develop diseases like hypertension, diabetes, coronary heart disease, metabolic disorders, pulmonary, renal and mental illnesses. The external environment affects the development of a particular phenotype suitable for an environment with characteristics that closely resemble intrauterine conditions. If the extra-uterine environment differs greatly from the intra-uterine one, the fetus is more prone to develop disease. Subsequent studies have shown that maternal diseases like depression and anxiety, epilepsy, asthma, anemia and metabolic disorders, like diabetes, are able to determine alterations in growth and fetal development. Similarly, the maternal lifestyle, particularly diet, exercise and smoking during pregnancy, have an important role in determining the risk to develop diseases that manifest themselves both during childhood and particularly in adulthood. Finally, there are abundant potential sources of pollutants, both indoor and outdoor, in the environment in which the child lives, which can contribute to an increased probability to the development of several diseases and that in some cases could be easily avoided.

## Introduction

Several epidemiological studies have revealed that exposure to an unfavourable environment in early life is associated with a significantly increased risk of later disease, a phenomenon termed *‘early life programming’*. Factors that adversely affect the gestational and early postnatal environment such as maternal diseases and their treatments, life-style such as nutrition and activity as well as exposure to pollutants can alter fetal development with persistent effects on health.

Major maternal causal diseases include psychiatric-neurologic disorders, diabetes, asthma, sleep related breathing disorders, and anemia. Furthermore, an increasing body of proof highlights the weight of a mother’s nutrition and of micro-nutrients, and life-style as well as exposure to pollutants, from preconception through lactation, in programming the emerging organ systems and homeostatic pathways of her offspring.

Moreover, exposure of the developing embryo or fetus to some environmental agents like gamma irradiation and thalidomide is well known to produce anatomical anomalies leading to *in utero* death or structural birth defects, commonly termed teratogenesis. Perhaps less well appreciated is that such environmental exposures also can cause functional disorders that persist postnatally and into adult life. This seems to be true also for hormones that when present in non-physiological concentrations during ‘critical periods’ of perinatal life can act as ‘endogenous functional teratogens’. For example perinatal hyperinsulinism, pathognomonic in the offspring of diabetic mothers, may lead to ‘malprogramming’ of neuroendocrine systems regulating body weight, food intake and metabolism. This results in an increased disposition to become obese and to develop diabetes throughout life [1]. However, the spectrum of such postnatal consequences is growing, and more recently is thought to include disorders of the immune system, brain function, and cancer, to name a few.

Many human teratogens elicit their deleterious effects through mechanisms involving the generation of reactive oxygen species (ROS) and oxidative stress [2]. Since many antioxidant regulation enzymes are not well expressed early in organogenesis, it may explain why embryos, in earlier periods of development, are more susceptible to teratogen-induced dysmorphogenesis and functional teratogenesis.

## Maternal diseases during pregnancy

### Maternal anxiety, depression and selective inhibitors of serotonin re-uptake inhibitors (SSRIs)

Over 13% of women experience episodes of depression during pregnancy or during the first year after the delivery and up to 18% of women develop anxiety syndromes [3]. This reflects on fetal development in different ways. Maternal depression during pregnancy increases the risk of delivering a baby of low birth weight but with a central distribution of adipose tissue, while post-partum depression is associated with an overall increase in fetal adipose tissue [4]. Depression in pregnancy influences the development of the hypothalamic-pituitary-adrenal axis by exposing it to higher concentrations of corticotrophin releasing hormone (CRH) which are related with lower body mass index (BMI) and higher central adiposity in the child [5]. Post-partum depression is associated with a reduction in breast feeding, which is a protective feature against the development of obesity; and a risk factor for unhealthy maternal behaviours like overeating and little exercise, that are behaviours inevitably transmitted to the child.

Moreover, children of mothers with high levels of anxiety-depressive disorders at 32nd week of gestation, have almost twice the risk (RR = 1.68) of developing asthma by the age of seven and a half years [6]. Nevertheless, exposure to maternal depression and anxiety restricted to the first year of life, has only a limited association with successive asthma (RR = 1.25) [7].

Another aspect that must be considered is the influence of maternal psychiatric disorders on the development and morphology of the fetal brain. Children whose mothers suffer from anxiety-depressive syndrome have less developed areas of the brain responsible for controlling cognitive functions, particularly the prefrontal cortex, the structure that regulates the schedule of action, reasoning, working memory, attention and some aspects of language [1]. Consequently the baby is exposed to an increased risk of a tendency to develop a less optimal mother-child interaction and insecure infant attachment [8] and having problem behaviours and lower competencies particularly in boys [9,10]. Further studies have shown that if the mother is depressed, the child has a three times greater risk (RR = 3.1) of developing attention deficiency and hyperactivity disorder (ADHD) [11,12], and almost twice the risk for both girls and children (RR = 1.91 and RR = 2.16, respectively) of developing changes in behaviour and emotional problems [13].

There might be several possible explanations for these findings but the most accredited one is the fact that during pregnancy, the placenta is a major extra hypothalamic site for CRH production and action. In contrast to the negative control exerted on the brain and pituitary gland, cortisol stimulates the production of CRH in the placenta, establishing a positive feedback loop that terminates upon delivery. The fetus is therefore exposed to high levels of glucocorticoids that affect the programming of the nervous system and make the child more prone to developing behavioural disorders [14].

Also the drug treatment of maternal depression may have long-term consequences on the child. Selective inhibitors of serotonin re-uptake (SSRIs) antidepressants interfere with the hypothalamus-pituitary-adrenal axis and with the circadian rhythms involved in fetal development as well as with some subtypes of serotonin receptors (5-HT-2B) responsible for the development of the fetal cardiovascular system [15,16]. For this reason, the use of paroxetine during the first trimester of pregnancy almost doubles (RR = 1.72) the risk of cardiac malformations [17], while drugs like fluoxetine, venlafaxine, sertraline and citalopram showed no such effect [18]. Exposure to these drugs after the 20th week of gestation increased six-fold the newborn’s risk (RR = 6.1) of persistent pulmonary hypertension. On the contrary, the use of SSRIs before the 20th week or the use of other antidepressants, were not associated with this risk [19]. The lung, in fact, might function as a reservoir for antidepressants because they accumulate there. Serotonin, in addition to the vasoconstrictor effect, function as a mitogen on the smooth muscle cells and inhibits the production of nitric oxide, a potent vasodilator during both intra-uterine and post-natal life [17].

Finally, exposure to SSRI antidepressants such as fluoxetine and paroxetine increases by three times the newborn’s risk (RR = 3) of antidepressant withdrawal syndrome. In this case, neonates primarily display central nervous system, motor, respiratory and gastrointestinal signs, that are usually mild and disappear within 2 weeks of age [20]. In fact, at the time of birth the concentrations of long half-life antidepressants, like paroxetine, gradually decrease. Furthermore, the potent inhibition of serotonin re-uptake and affinity for muscarinic receptors may be responsible for the withdrawal syndrome and excessive muscarinic stimulation [18].

### Epilepsy and antiepileptic drugs in pregnancy

Epilepsy is a frequent neurological disorder affecting 0.4-0.8% of pregnant women. Women with epilepsy have a higher risk of preeclampsia, gestational hypertension, bleeding in pregnancy and excessive bleeding postpartum. They also have higher incidence of congenital anomalies and delayed cognitive development in their children. It has been uncertain if the increased risk of complications is due to the epilepsy per se, the use of antiepileptic drugs, or the combination of both factors [21]. Recent studies powerfully point to an association to the medications and the dose used in pregnancy. The use of anti-epileptic drugs during pregnancy has been associated with the development of anticonvulsant embryopathy that comprises the development of major malformations, growth retardation and hypoplasia of the midface and fingers. The frequency of malformations is directly proportional to the number of drugs taken by the mother: monotherapy almost triple (RR = 2.8) the risk of embryopathy whereas polytherapy (taking more than two drugs) increases the risk over four times (RR = 4.2) [22]. Children of epileptic mothers who did not take drugs during pregnancy had no increased risk of embryopathy compared to controls. Therefore, according to Cochrane’s guidelines, women with a history of epilepsy should continue medication during pregnancy using monotherapy at the lowest dose required to achieve seizures control. When it’s possible, women should avoid polytherapy (this is the practice in 40% of women) [23]. In particular, valproate should not be used as a drug of choice during pregnancy because it is responsible for an increased risk of impaired cognitive function in children at 3 years of age. This risk is dose-dependent. Therefore, valproate should not be used a first choice medication in women of childbearing prospective [24].

### Type 2 diabetes

Fifteen percent of pregnant women develop impaired glucose tolerance and more than 5% of these develop diabetes [25]. Vigorous physical activity (7 to 13 hours weekly) before pregnancy and light to moderate (3 to 6 hours) or vigorous exercise during pregnancy may reduce the risk of both impaired glucose tolerance and diabetes mellitus in pregnancy by approximately 50% and 30%, respectively [23]. Skeletal muscle contraction, in fact, triggers glucose uptake and promotes insulin sensitivity, and more intense exercise has a stronger hypoglycemic effect [26]. Maternal hyperglycemia and obesity, in fact, expose the fetus to hyperinsulinemia and to a higher deposition of fat, with consequent increased risk for developing metabolic syndrome. The risk of this disease doubles for children born large for gestational age (RR = 2.19), is almost double for those born to obese mothers (RR = 1.81) and then increases by one and a half for those born to mothers with diabetes mellitus (RR = 1.44). Thus, the relationship between prenatal nutritional status and metabolic diseases is shaped like a U as the risk increases at both ends of the birth weight curve *i.e.* in conditions of poor nutrition and excessive dietary intake [27]. This relationship can be clearly showed in the population of the Pima Indians. The Pimas successfully adapted to the desert life by elaborating an irrigation system and by hunting to supplement their cultivated crops, but by the end of the 19th century European people disrupted their traditional agriculture and led to important changes in their way of life. The Pima Indians now have the world’s highest prevalence and incidence of type 2 diabetes [28], a relative new condition with 70% of 35 years old subjects with type 2 diabetes. The effects of the diabetic pregnancy can be thought of as a vicious cycle. The woman with diabetes, diagnosed before or during pregnancy, has a high-risk pregnancy with potential complications extend well beyond the neonatal period. The infant of the woman with diabetes is at high risk of becoming obese and of developing Type 2 diabetes at a young age. The young woman whose mother had diabetes during pregnancy is at risk of perpetuating the cycle by becoming obese and developing diabetes before or during her childbearing years [29]. Moreover, metabolic conditions, like obesity, are associated with a higher likelihood of autism spectrum disorders (OR = 1.61) and developmental delays (OR = 2.35) [30]. In fact, in a diabetic and possibly prediabetic pregnancy, poorly regulated maternal glucose can result in adverse fetal development. Prolonged fetal exposure to elevated glucose levels results in chronic fetal hyperinsulinemia, with a consequent trigger to increased oxygen consumption and metabolism, leading to chronic intrauterine tissue hypoxia [31]. This can profoundly affect neurodevelopment, including alterations in myelination and cortical connectivity and aberrations in hippocampal neurons [32].

**Figure 1:**
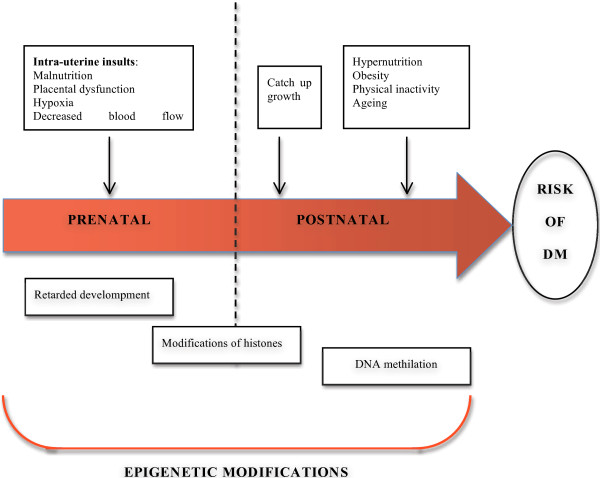
Prenatal and postnatal factors contributing to the development of diabetes mellitus.

Epigenetic events also play a role in determining susceptibility to metabolic diseases. Intrauterine stressors, including poor maternal nutrition and placental dysfunction (alteration in the flow of nutrients and hypoxia), may affect the development and cause epigenetic modifications. Additional environmental factors in the postnatal period, including accelerated postnatal growth, obesity, inactivity and aging, further contribute to the risk of diabetes mellitus probably through modifications and DNA methylation in critical tissues (Figure 1) [33].

### Asthma and its treatment in pregnancy

Nearly 10% of pregnant women are affected by asthma, the disease that most commonly complicates the course of a pregnancy. The recognition and optimal control of this disease is crucial for both mother and fetal well being. Asthma exacerbations during pregnancy are associated with low birth weight, especially in males [34]; whereas inadequate control of symptoms can lead around an 11% incidence of preterm delivery compared with 6% of preterm delivery in well controlled cases. The incidence rises to over 16% if hospitalization is required. These effects seemed independent from use of systemic corticosteroids [35]. A pregnant mother’s poorly controlled asthma is risky for both mother and fetus, much more than the side effects of the drugs used to control symptoms [27,36].

The use of short and long acting ß_2_ agonists, inhaled corticosteroids such as budesonide [37] or oral corticosteroids, do not increase the risk of malformations, while the use of chromones in pregnancy increases the incidence of malformations of the musculoskeletal system [38].

Furthermore, one epidemiological study has shown that forty percent of subjects presents one or more risk factors such as: maternal or paternal asthma or asthma during childhood, maternal smoking and respiratory infections during childhood. Those individuals have a higher risk of reduced forced expiratory volume in 1 second (FEV_1_) between the ages of 29 and 44 years. The presence of 3 or more of these factors increases the risk of developing adult chronic lung disease or obstructive pulmonary disease (COPD) sixfold for males (RR = 6.3) and sevenfold for females (RR = 7.2) [39]. Thus, chronic obstructive pulmonary disease (COPD) find its origins in childhood but manifest itself in adulthood. The above factors together with cigarette smoking confer a risk of COPD which is higher than just smoking [40].

### Breathing disorders during sleep or sleep deprivation during pregnancy

**Figure 2:**
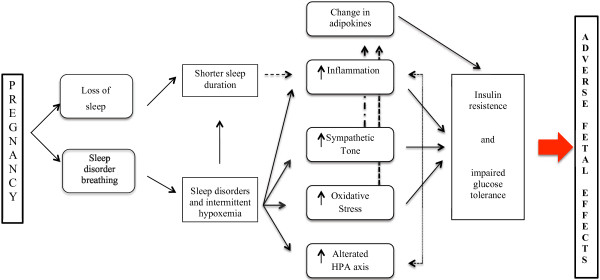
Seep disorders during pregnancy influence the risk of insulin resistence and impaired glucose tolerance.

There is a number of breathing disorders during sleep, ranging from simple snoring to severe forms of obstructive sleep apnea syndrome and obesity-hypertension. During the third trimester, when gestational sleep disorder breathing (SDB) is most likely to arise, the prevalence of habitual snoring has been evaluated to be present in between 10% and 27% of pregnant women [41]. About 7% of women with respiratory disorders during sleep delivers a baby small for gestational age, with a more than threefold increased risk (RR = 3.45), or a baby with low Apgar scores and a higher risk of mortality [42]. SDB are associated with gestational diabetes with a risk 2 to 7 times higher than the controls with pathogenetic mechanisms depicted in Figure 2[43,44]. The onset of hypertension is associated not only with maternal morbidity but also with increased fetal mortality or subsequent risk of development of hypertension, stroke, metabolic syndrome and premature death from cardiovascular causes [45]. Furthermore, sleep disorders, given the poor cardiopulmonary reserve of the mother generate a state of hypoxemia resulting in decreased delivery of oxygen to the fetus, rapid onset of respiratory acidosis and fetal bradycardia [46].

### Anemia in pregnancy

Anaemia of pregnancy is defined as haemoglobin (Hb) <110 g/L or 115 g/L in some clinical practice guidelines with a slight variation according to the trimester of pregnancy. However, an haemoglobin level <100 g/L denotes anaemia at every stage during pregnancy that should be investigated and treated because of possibly serious effects for the mother and her baby, with an increased risk of intrauterine growth retardation and premature birth [47,48]. One of the most frequent causes of anaemia is iron deficiency [49]. Young women and particularly pregnant women are at high risk for iron deficiency [41]. Though such problem is mainly present in developing countries, it also affects a high percentage of women of reproductive age (10-30%) in the industrialized world [50]. Ferritin level is considered the surrogate marker for iron deficiency, which can be classified as severe when the serum ferritin level is below 20–30 *μ*g/L and mild-moderate if the serum ferritin level is below 70–100 *μ*g/L [40]. One of the reasons for iron deficiency, could be the low intake of iron from the diet [51]. This problem could be aggravated in pregnancy because during this period iron demand is increased in order to cover the requirements of the rise of the red cells mass, the expansion of the plasma volume and to allow for the growth of the feto-placental unit [52]. Generally, iron deposits derived from the mother are exhausted within the first 6 months of life, after which the child must begin to produce iron independently. Although in some cases babies born to mothers with anemia may have normal levels of iron, because of active placental iron transport; however levels are typically lower than infants born to mothers without anemia. It has been shown that preconceptional [53] and early pregnancy anaemia [54] are associated with an increased risk of low birth weight, birth length and head circumference. Several mechanisms have been proposed to clarify the pathways through which iron deficiency and anemia could weaken fetal growth. Iron deficiency and hypoxia resulting from anemia can induce maternal and fetal stress thus increasing norepinephrine concentration. The stress, in turn, activates the production of corticotrophin-releasing hormone which stimulates fetal cortisol. As a result, longitudinal growth of the fetus could be impaired by the action of cortisol. Another possible explanation is that iron deficiency causes oxidative damage on erythrocytes and the feto-placental unit. Iron deficiency can also increase the risk of maternal infections, which can stimulate the production of CRH and are a major risk factor for preterm delivery [55].” Moreover, offspring of mother with anemia had doubled risk of wheezing (OR = 2.42) and more than triple the risk of current asthma at the age of six years (RR = 3.46) [56]. On the basis of such evidences, the fact that low to moderate dose iron supplementation in early pregnancy benefits fetal growth in women with iron deficiency is not surprising. The recommended daily dietary allowance for iron in pregnancy is 27 mg instead of 8 mg in the adult non pregnant population. Lactation requires a daily dietary allowance of 10 mg [57,58]. The positive effect on fetal growth could be explained by a preferential transfer of iron to the placenta and fetus [58]. Even the lack of other micro-nutrients such as selenium, vitamins E, D and C, zinc and folic acid is responsible for the onset of disorders affecting the respiratory system in the offspring [46].

## Maternal lifestyle and environment

### Maternal behaviour, interpersonal stress and psychosocial trauma

Maternal behaviour during early periods of life can alter the epigenetic state of the fetal DNA. Studies in mouse models have shown that infants rats whose mother had a high frequency of “licking, grooming and arched-back nursing” are able to better react to stress [59]. Maternal care during infancy regulates the development of neural systems mediating the expression of fearfulness in the rat. In fact, it has been showed an increased hippocampal expression of the glucocorticoid receptor mRNA and protein, a decreased hypothalamic corticotropin release factor and reduced hypothalamic-pituitary-adrenal response to stress in pups born from mothers with licking, grooming and arched-back nursing behaviour [60,61].

Maternal stress and maternal-placental-fetal biological mediators of stress can affect fetal development. In fact it has been showed that exposure to maternal psychosocial stress during intrauterine life is associated with significantly shorter leukocyte telomere length in young adulthood, a predictor of age related disease onset and mortality [62]. This provides a biological basis for speculation about the effects of early occurrence of poverty and how exposure to abuse, family conflict, emotional neglect and severe discipline could lead to individual differences in the nervous and endocrine response to stress and increase susceptibility to common adult disorders such as depression, anxiety, drug abuse or chronic diseases like diabetes, cardiovascular disease and obesity.

Moreover, stress during pregnancy is associated with the risk of delivering a preterm baby or a child small for gestational age [63,64] and it contributes to less control of the parasympathetic system on heart rate, with a low heart rate variability in response to stressful stimuli [65]. In a study it has been shown that prenatal anxiety and stress predicted a substantial amount of variance in infant diseases and antibiotic use and precisely 9.3% for respiratory, 10.7% for general, 8.9% for skin, and 7.6% antibiotic use [66]. Moreover, maternal stress during pregnancy alters the cytokine response of the innate and adaptive immune system. If on the one hand high levels of IL-8 and TNF-α in response to microbial stimuli are observed, on the other, there is a reduction in levels of IFN-γ with an increase in IL-13. This generates an obvious imbalance of the immune response in favour of Th2 lymphocytes and susceptibility to allergic diseases [67,68]. An association between prenatal stress and immune function in human adults has been documented. Peripheral blood mononuclear cells from healthy young women whose mothers experienced major negative life events during their pregnancy (Prenatal Stress, PS group), and from a female comparison group, were stimulated with phytohemagglutinin (PHA), and subsequent cytokine production was measured. A bias for T-helper 2 (Th2) cytokine production due to an overproduction of IL-4 relative to IFN-gamma after PHA stimulation was observed in PS subjects. In addition, IL-6 and IL-10 were also significantly elevated suggesting a direct association between prenatal stress exposure and alterations in immune parameters in adult women [69]. In fact, children whose mothers experienced interpersonal violence and trauma during pregnancy have twice the risk of developing asthma during their childhood [70].

Exposure to maternal stress also affects the child’s cognitive performance such as language, comprehension and reasoning, all of them principally realized in the prefrontal cortex. This brain region is known to develop later in term of myelination and synaptic density. Because of its protracted development and the expression of glucocorticoid receptors, the prefrontal cortex may be prone to early insults. Since children exposed to high levels of hydrocortisone have longer time reactions, this could provide support for an association between prenatal stress exposure and the potential modulatory effect of cortisol on the working memory performance, which may reflect compromised development of the prefrontal cortex [71].

Finally, prenatal stress increases the future risk of insulin resistance, with higher levels of insulin and C-peptide than in controls as well as a lipid profile compatible with the development of metabolic syndrome [72].

### Smoking in pregnancy

Cigarette smoke contains more than 4000 compounds, including polycyclic aromatic hydrocarbons such as arilamine and N-nitrosamines. The ability of the individual to convert these substances into less toxic compounds is important to minimize their adverse effects. This is made possible by enzymes that allow the metabolism and detoxification of these substances. Enzymes such as CYP1A1 and glutathione-S-transferase (GSTT1), important for the detoxification of the compounds, are involved in this process. Polymorphisms of these genes have been associated with a reduction in birth weight of between 250 g and 600 g [73], with a higher risk of delivering a baby of low birth weight (< 2500gr) (RR = 1.32) or small for gestational age (RR = 1.21) [74], and a linear dose–response relationship between exposure and second trimester femur growth was observed with almost 1 cm lower femur growth for the highest versus the lowest tertile of exposure [75]. However, the side effects of smoking may be reversible in early pregnancy because women who quit smoking before the 15th week of gestation have a lesser probability of having a premature baby or one small for gestational age, which is not different from that of non-smoking women, 4% and 10%, respectively. Conversely, if a woman continues to smoke, the incidence of preterm birth is around 10% and the risk of having a baby small for gestational age raises at 17% [76]. Furthermore, children exposed to cigarette smoke continue to have respiratory problems during childhood, with twice the risk (RR = 2.18) of having lower values of FEV_1_[75]. In a systematic review and meta-analysis it has been shown that exposure to pre- or postnatal passive smoke exposure was associated with a 30% to 70% increased risk of incident wheezing (strongest effect from postnatal maternal smoking on wheeze in children aged ≤ 2 years, OR = 1.70) and a 21% to 85% increase in incident asthma (strongest effect from prenatal maternal smoking on asthma in children aged ≤ 2 years, OR = 1.85) [77]. Maternal smoking during pregnancy leads to abnormal lung function in infancy that tracks through to later childhood and continues into adult life. This is associated with transient wheezing illnesses through early childhood and increases the risk of chronic obstructive pulmonary disease in the elderly [78]. Also passive exposure of pregnant women to ETS may lead to asthma in their offspring: passive exposure to ETS, mainly during the third trimester of pregnancy, was significantly associated with asthma- and allergy-related symptoms after adjusting for several confounders in a multivariate analysis (current wheeze: OR = 1.42, pruritic rash ever: OR = 1.45). As a consequence public health policies should be oriented not only towards smoking cessation, but also reinforce elimination of ETS exposure of pregnant women [79]. In brief, exposure to passive smoking increases the incidence of wheeze and asthma in children and young people by at least 20%. Preventing parental smoking is crucially important to the prevention of asthma. Nicotine, in fact, causes higher placental vascular resistance, decreases blood flow in the uterus, and increases the concentration of carboxyhaemoglobin, all factors responsible for chronic hypoxia and reduced fetal development [80]. In addition, children of smoking mothers have a higher risk (RR = 1.5) of being overweight or obese [81,82] because nicotine withdrawal promotes overeating and weight gain, and on the other, children born from smoking mother tend to get less exercise and have a lower quality diet [74].

Children exposed to tobacco smoke during pregnancy also have a higher risk of poor neurodevelopmental outcome [80]. In a prospective follow-up study of infants with a birth weight ≤ 1500 g or a gestational age < 32 weeks, the brain was imaged by serial brain ultrasound examinations until discharge and magnetic resonance imaging at term age and it has been found that the frontal lobe (P = 0.01) and the cerebellar (P = 0.03) volumes were significantly smaller in the exposed than in the unexposed infants [83]. This is consistent with reports showing an association between prenatal smoking exposure and impairments in frontal lobe and cerebellar functions such as emotion, impulse control, and attention. Effectively, these children have a more than doubled risk of developing attention deficit disorder and hyperactivity disorder (ADHD) (RR = 2.5) [84,85] compared to babies born to non-smoking mothers. Genes relating to the dopaminergic pathways are the focus of most genetic studies of AHD. The gene for the dopamine transporter (DAT) is of particular interest because is the site of action of psychostimulants. It has been shown that homozygosis for this gene and exposure to cigarette smoke during pregnancy increase the risk of developing ADHD and determine a poor response to psychostimulants [86]. This risk is approximately three times greater when the gene for the dopamine transporter DAT1 and subtype D4 for the dopamine receptor DRD4 is inherited (RR = 2.9 and RR = 3, respectively) but inheriting both alleles increases by 9-fold the risk of ADHD (RR = 9) [79].

### The role of the home

The home is the place where children spend most of their time, estimated at 15 hours out of 24 [87]. Given the stable increase in the incidence of asthma among children, it is important to pay attention and implement environmental measures in the home to help prevent it. Contact with synthetic textiles should be avoided in the first ages of life: during the first months of life sleeping in synthetic sleeping bags increases by more than 4 times the risk (RR = 4.33) of wheezing at the age of seven years [88]. Furthermore, the use of synthetic pillows doubles the risk (RR = 2.5) of wheezing and the risk rises to more than five times (RR = 5.2) when both synthetic pillows and blankets are used [89]. Synthetic materials, in fact, have a structure that allows the accumulation of high concentrations of dust mite allergen (Der p1), even 15 times higher compared to cotton fabrics [90]. Furthermore, synthetic pillows contain significantly more pet allergens than feather pillows, supporting the view that tightly woven encasements surrounding feather pillows act as a barrier for allergens [91].

In addition, chemicals used for cleaning are responsible for increasing domestic pollution and more than doubling the risk of wheeze (RR = 2.3), [92] and they cause a decrease in FEV_1_ and FEF_25-75_[93]. This derives from both a detrimental effect on the development of airways in the prenatal period (epigenetic effect?) but also from the effects in the post-natal period. In particular, this association was noted for the use of cleaning sprays [85] that contain active ingredients such as alcohol, ammonia, chlorine, glycol and glycol-ethylene, sodium hydroxide (caustic soda), acrylic polymers and terpenes [94]. When sprayed on surfaces to be cleaned, these compounds enter easily into the airways, irritating them [86].

### The role of environmental pollution

In New York City it has been shown that prenatal exposure to pollutants doubles the risk of delivering an infant small for gestational age and increases by five times the risk of preterm delivery [95]. These effects were observed in African-American but not in Domenicans and this may reflect modification of the risk by healthful cultural practice among Domenican immigrants including diets with higher nutritional quality and greater social support. In the same environment it has been reported that infants of pregnant women exposed to high concentrations of polycyclic aromatic hydrocarbons have more than twice the risk of developing cognitive delay at age three, resulting in poor school performance and low IQ at age 5. Furthermore, forty percent of the analyzes on the DNA of white blood cells of umbilical cord blood of New York City children showed damage to the DNA reflecting transplacental exposure to traffic-related polycyclic aromatic hydrocarbons (PAHs). The increased formation of DNA adducts is associated with a possible consequent increased risk in adulthood of cancer [96] and of asthma [97]. At this regard it has been shown that for concentrations of 2.41 ng/m^3^ of PAHs the risk of finding methylation in the DNA increases by almost 14 times (RR = 13.8) and the risk of developing asthma increases by almost four times (RR = 3.9). Concerns about pollutants were always related to elderly people and respiratory diseases, but now we start to understand that fetal tissues are exposed as well with long time persistent consequences. Based on these observations, the reduction of environmental pollutants, mostly from vehicle fuel combustion, could help reduce the incidence of several diseases.

### Dietary recommendations

A pregnant mother’s diet has both an immediate protective effect, and a long lasting effect on life. We have discussed the fact that exposure to pollutants like PAHs increases the risk of mutations, with DNA adduct formation and carcinogenesis. However it has been shown that antioxidants protect against damage caused by these substances. Subjects with low levels of alpha tocopherol have an almost four times higher risk (RR = 3.96) of having high levels of DNA adducts than those with high levels of antioxidants [98]. Also fish consumption during pregnancy contributes to reduce the risk of low birth weight [99], preterm birth (<34 weeks), the number of low birth weight babies and the number of admissions to neonatal intensive care [100]. Other studies demonstrated the protective role of fish against the development of atopic dermatitis at the age of one year and asthma at six years, with a decrease of 30% and 50% in the incidence of these allergic diseases, respectively [101].

It has been reported that women who consumed high amounts of fish during pregnancy had a lower incidence of postpartum depression during the first six months after delivery (9.67% vs. 11.19% of women with low intake), while not all authors agree on the protective role on the development of the brain and cognitive functions [92,102]. Fish is an important source of polyunsaturated fatty acids (docosahexaenoic acid DHA-EPA-and acid-eicosaepentanoic-) and essential nutrients that are not produced by the human body, and which play a protective role in the development of the neurological, immune and cardiovascular systems. They are essential for the development of the brain and retina, tissues that have the maximum uptake of these substances during the second half of pregnancy and early in childhood. However, not all types of fish can be consumed during pregnancy, because in some kind of fish the content of mercury is higher. Salmon, shrimp and hake are recommended because they are rich in ω-3 fatty acids and low in mercury, whereas large deep-water fish like tuna, swordfish and Atlantic shark should be avoided [103,104]. Small-sized fish, like sardines can be consumed because of their high content of selenium, an element capable of reducing the toxicity of mercury [105].

A pregnant mother’s diet that includes fruits and vegetables can also prevent asthma and atopic dermatitis [106]. Furthermore, levels of maternal vitamin E affect the development of fetal biometrics (crown-rump length, femur length and biparietal diameter) and lung development: for every millimeter of increase in fetus length, there is an increase of up to 5 ml of FEV_1_ and 6 ml of FVC [107]. Intake of vitamin D during pregnancy, either through the diet or through supplements, has a protective role against asthma and atopic dermatitis. High levels of maternal vitamin D can reduce the risk of asthma and wheezing in children by up to 60% [108,109]. The average dose of vitamin D to be taken is about 600 UI/day [110], but many studies indicate that a higher dose of vitamin D during pregnancy and lactation, up to 1000 UI/day, is necessary to achieve a good level of vitamin D assets [111].

Finally, maternal cholesterol levels below the 10th percentile (<159 mg/dl) and higher than the 90th (261 mg/dl) are associated with an increased risk of preterm delivery. Children born at term to mothers with low levels of cholesterol weigh about 150 grams less than controls, and have a higher risk of microcephaly [112]. Cholesterol Low-density lipoprotein (LDL) represents the main substrate for the synthesis of progesterone and cell membranes of the decidua, essential for proper installation and vascularization of the placenta. Alterations in placental cholesterol concentrations cause changes in placental transport functions and fetal growth retardation [104].

### Folic acid and pregnancy

According to the U.S. Preventive Services Task Force all women of childbearing age or who are planning to become pregnant should take daily supplements of folic acid, at a dose range between 0.4 and 0.8 mg, to prevent neural tube defects. Several studies provide evidence that specific genes and DNA methylation sites are subject to change during development and during a lifetime as a direct response to nutrition. Studies of the methyl donors-folate, choline, and methionine offer the most convincing evidence of a role in mediating DNA methylation changes [113]. Folic acid is essential for the synthesis and function of DNA and affects the embryogenesis of the nervous system [114]. Since a requirement for folic acid intake during pregnancy was introduced, there has been a 19% reduction of neural tube defects (anencephaly and bifid spine). However, only about 35% of women of childbearing age take the minimal daily dose of folic acid, and annual consumption of folic acid is decreasing [115]. Supplementation with acid folic decreases the incidence of the risk of preterm delivery in the period 20-28th week of gestation by 70% and by 50% during the 28-32nd week period; but has no influence on the labor thereafter. In fact, low concentrations of folic acid can alter the function of lymphocytes and neutrophils, increasing the risk of bacteriuria in pregnancy, which in turn can increase the risk of preterm delivery [116].

## Conclusions

Studies in both animal and human models have shown that exposure to particular events during the critical stages of pregnancy can alter the expression of genes in the fetus. This leads to changes that may persist throughout life causing increased susceptibility to disease. Adverse events that lead to embryonic, fetal or neonatal epigenetic changes are responsible for altering the mechanisms of growth and metabolism observed later in childhood. The health of the child and issues that will determine its appetite and metabolism, intelligence and temperament in life depend on the type and amount of nutrients that the baby receives in the womb, pollution, drugs and infections which it is exposed to before and after birth, the mental and physical health of the mother and her levels of stress and mental illness.

## Competing interests

The authors declare that they have no competing interest.

## Authors’ contributions

All authors read and approved the final manuscript.
